# Age related retinal Ganglion cell susceptibility in context of autophagy deficiency

**DOI:** 10.1038/s41420-020-0257-4

**Published:** 2020-04-17

**Authors:** Katharina Bell, Ines Rosignol, Elena Sierra-Filardi, Natalia Rodriguez-Muela, Carsten Schmelter, Francesco Cecconi, Franz Grus, Patricia Boya

**Affiliations:** 1 Department of Cellular and Molecular Biology, Centro de Investigaciones Biológicas Margarita Salas, CSIC, Madrid, Spain; 2 Experimental and Translational Ophthalmology, Medical Center of the Johannes Gutenberg University, Mainz, Germany; 3 Deutsche Zentrum für Neurodegenerative Erkrankungen e.V, DZNE/German Center for Neurodegenerative Diseases, Dresden, Germany; 4 Department of Biology, University of Rome Tor Vergata, 00133 Rome, Italy

**Keywords:** Cell death in the nervous system, Mitophagy

## Abstract

Glaucoma is a common age-related disease leading to progressive retinal ganglion cell (RGC) death, visual field defects and vision loss and is the second leading cause of blindness in the elderly worldwide. Mitochondrial dysfunction and impaired autophagy have been linked to glaucoma and induction of autophagy shows neuroprotective effects in glaucoma animal models. We have shown that autophagy decreases with aging in the retina and that autophagy can be neuroprotective for RGCs, but it is currently unknown how aging and autophagy deficiency impact RGCs susceptibility and survival. Using the optic nerve crush model in young and olWelcome@1234d *Ambra1*^+/gt^ (autophagy/beclin-1 regulator 1^+/gt^) mice we analysed the contribution of autophagy deficiency on retinal ganglion cell survival in an age dependent context. Interestingly, old *Ambra1*^+/gt^ mice showed decreased RGC survival after optic nerve crush in comparison to old *Ambra1*^+/+^, an effect that was not observed in the young animals. Proteomics and mRNA expression data point towards altered oxidative stress response and mitochondrial alterations in old *Ambra1*^+/gt^ animals. This effect is intensified after RGC axonal damage, resulting in reduced oxidative stress response showing decreased levels of *Nqo1*, as well as failure of *Nrf2* induction in the old *Ambra1*^+/gt^. Old *Ambra1*^+/gt^ also failed to show increase in *Bnip3l* and *Bnip3* expression after optic nerve crush, a response that is found in the *Ambra1*^+/+^ controls. Primary RGCs derived from *Ambra1*^+/gt^ mice show decreased neurite projection and increased levels of apoptosis in comparison to *Ambra1*^+/+^ animals. Our results lead to the conclusion that oxidative stress response pathways are altered in old *Ambra1*^+/gt^ mice leading to impaired damage responses upon additional external stress factors.

## Introduction

Glaucoma are a group of diseases leading to progressive optic nerve damage and accompanying visual field loss, caused by retinal ganglion cell (RGC) death. This multifactorial, neurodegenerative disease is one of the leading causes for blindness and although estimating worldwide prevalence for all glaucoma cases is challenging, we know that glaucoma will affect at least 111.8 million people by the year 2040^1^. The risk for developing glaucoma increases with age and prevalence rapidly rises after the age of 40 years^1^. Next to the clinically most relevant risk factors such as increased intraocular pressure and ageing, one major factor associated with RGC death in glaucoma is mitochondrial dysfunction, which was recently confirmed in a proteomics study analysing human retinal post-mortem glaucoma samples^2–4^.

Autophagy is an important cell catabolic pathway necessary for the recycling of intracellular components via their delivery to the lysosome^5^. During autophagy the material targeted for degradation is packed into a double membrane structure, the autophagosome, which then can fuse with the lysosome for cargo degradation^6^. Not only soluble cell fractions such as proteins are degraded via autophagy, but also organelles, such as mitochondria, during selective autophagy. In this process cargo receptors and adaptors determine specificity for organelles and other cargo for degradation^7^. Autophagy levels decline with age in many tissues including the brain^8–11^ which contributes to the pathogenesis of various, if not all age-related neurodegenerative diseases^12–14^. Our data in the aging retina also supports this notion and we find decreased mRNA expression of several autophagy regulators such as *Atg7* and *Beclin1* that results in decreased autophagy flux from 12 months of age onwards in the mouse retina^11^. Other studies have demonstrated the importance of autophagy for proper neuronal development and embryonic survival^15,16^. Programmed cell clearance in early stages of retinal development is dependent on autophagy^17^ and we have shown that mitophagy is important for RGC development, leading to the metabolic shift towards glycolysis which is essential for retinal ganglion cell neurogenesis^18^. Genetic studies show a link between glaucoma and autophagy related genes^19^. Of the genes that have been found associated with glaucoma in genome-wide association studies (GWAS) studies *Optn*^20^, *Tbk1*^21^, and *Opa-1*^22–24^ play a role in mitochondrial function and also in mitophagy pathways^25–28^. Interestingly, these genes are supposed to play a role in normal tension glaucoma (NTG) rather than primary open angle glaucoma (POAG), which is, in contrast to NTG, associated with elevated intraocular pressure. Approximately 30–40% of glaucoma patients suffer from NTG^29^.

In this study we analysed the effect of ageing and autophagy deficiency on RGC survival using *Ambra1*^+/gt^ mice. AMBRA1 (autophagy/beclin-1 regulator 1) plays a role in the initiation of the autophagy process^30^. It also is a relevant mitophagy receptor that can act in a PINK1/PARKIN dependent and independent way^31–33^. *Ambra1*^gt/gt^ mice display embryonic lethality at E10–E14.5 associated with significant neural tube defects^30^. *Ambra1*^+/gt^ animals have been used as a model of subtle autophagy deficiency in many studies including our own, where we show alterations in differentiation of neural stem cells^34^. In this study we find that aged *Ambra1*^+/gt^ mice have increased susceptibility to axonal insult in vivo in a model of optic nerve crush (ONC). Proteomic analysis shows mitochondrial alterations and changes in the oxidative stress response in the *Ambra1*^+/gt^ animals that could increase the susceptibility to RGC damage.

## Materials and methods

### Animals procedures and optic nerve crush (ONC) surgery

5–6 months (young) and 12–14 months (old) *Ambra1*^+/+^ as well as *Ambra1*^gt/+^ mice with a CD1 background were used for these experiments. The ratio male/female in all experiments was 1:1 and we could not detect any apparent changes in behaviour of the female mice in our colony. Animals were housed, cared for and euthanized in accordance with European Union guidelines and experiments were approved by the CIB ethics committee for animal experimentation. The mice were housed in a 12 h/12 h light/dark environment and fed ad libitum. AMBRA1 mutant mice were provided by Francesco Cecconi, University of Rome Tor Vergata^30^. The animals were genotyped by processing tail bud tissue. DNA was isolated from tissue using the NzyTech gDNA Isolation kit (NZYTech) and PCR was performed as previously described^30^. In preparation for the ONC the mice were anaesthetised with a weight adapted intraperitoneal (i.p.) injection of 80 mg/kg ketamine chlorhydrate solution (Merial, Barcelona, Spain) and 10 mg/kg 2% xylacine chlorhydrate (Bayer, Barcelona, Spain). For surgery, the body temperature of the animals was maintained by placing them on a 37.5 °C heating platform.

ONC was performed on the left eye. Using 2.5 mm Vannas Spring Scissors (Fine Science Tools), the conjunctiva and tenon was opened from the 1 o’clock to the 3 o’clock position. By slightly rotating the eyeball nasally and using fine forceps the optic nerve was carefully exposed. Using self-closing negative action tweezers (Dumoxel Dumont Negative Action Tweezer, Style N7) the optic nerve was crushed for 7 s at a site between 1 and 2 mms behind the globe. During surgery both eyes were moisturised with artificial eye drops, which was continued on the non-surgery eye until the animals woke up. After surgery Ofloxacin eye ointment was applied to the left, surgery eye and all mice received an intraperitoneal injection of Buprenorphine (Buprenorphine; Bedford Laboratories, Bedford, OH, USA). Depending on the experiment, the mice were sacrificed 3, 4, 7, or 10 days after the ONC (indicated for each experiment) via cervical dislocation after a brief isoflurane sedation.

### Histological evaluation

The eyes were enucleated after cervical dislocation. For flatmount preparation, the eyes were opened through the cornea, the lens and vitreous body were extracted. Four petal cuts were performed in the retina, which was then mounted on nitrocellulose with the photoreceptors facing down. After fixing in 4% PFA 1 h at RT and washing with PBS, half of the flatmount was used for RGC counts and the other half of the retina was embedded in OCT and cryosections were prepared as previously described^35^. For RGC counts, the flatmounts were stained for Gamma-Synuclein (Abnova, H00006623-M01; 1:100, RGC marker) and Calbindin (Sigma, C2724; amacrine cells, 1:100) over night at 4 °C in a humid chamber. Brn3a staining in the cryosections was performed as previously published^36^. To stain RGCs with Brn3a in the cryosections, the sections were re-fixed with 4% PFA for 10 min, washed 3× with PBS for 10 min, then treated with 10 mM citrate buffer pH 6.0, heated in the microwave, and subsequently permeabilized with 1% Triton 4 × 20 min. After washing, the sections were blocked with BGT (0.3% BSA, 0.75% glycine, 0.25% triton X-100) for 1 h and then the primary antibody (anti-Brn3a, Millipore, MAB1585) was incubated over night at 4 °C. The following day, appropriate secondary antibodies (Molecular Probes, anti-rabbit, and anti-mouse) were incubated for 1 h at RT. The flatmounts were mounted in Fluoromount-G (Cultek, 100-01) with DAPI 1:1000 (D9542, Sigma).

Pictures of the ganglion cell layer of the flatmounts as well as the cryosections were taken with a confocal microscope (TCS SP5; Leica Microsystems, Barcelona, Spain). Masked cell counting was performed using the Image J software (NIH, Bethesda, MD, USA) on maximal confocal projections. For the flatmounts 4–6 fields per half retina were acquired with a X63 objective. Hereby the cells only stained with Gamma-Synuclein were accounted as RGCs. For the Brn3a staining in the cryosections 12–16 pictures from eight sections covering all regions of the eye were taken per retina.

### Quantitative RT-PCR

Retinal mRNA levels were measured from 13-month-old *Ambra1*^+/gt^ and *Ambra1*^+/+^ 3 and 7 days after ONC (*n* = 4–5 per group). Total RNA from retinas was extracted using TRIzol reagent (Invitrogen), and reverse transcription performed using the High‐Capacity cDNA Reverse Transcription Kit (Applied Biosystems, Waltham, MA, USA) according to the manufacturer’s instructions. Quantitative real‐time PCR was performed with 10 ng cDNA in a Light Cycler^®^ 480 Instrument (Roche, Mannheim, Germany) with LightCycler^®^ 480 probes master mix (Roche, Mannheim, Germany) using Taqman assays (Life Technologies, Carlsbad, CA, USA). TaqMan Gene expression arrays for *Mfn2* (Mm00500120_m1), *Optn* (Mm01333245_m1), *Park2* (Mm00450187_m1), *Pink1* (Mm00550827_m1), *Gpx1* (Mm00656767_g1), *Nqo1* (Mm01253561_m1), *Bnip3* (Mm01275600_g1), *Bnip3l* (Mm00786306_s1), *Nfe2l2* (Mm00477784_m1), *Becn1* (Mm01265461_m1). Assays were performed in duplicate, and results were normalised according to expression levels of 18s RNA (Hs99999901_s). Ratios were calculated using the contralateral eye.

### Proteomics

Retinae from 13-month old *Ambra1*^+/gt^ and *Ambra1*^+/+^ animals were isolated and stored at −80 °C until further use (*n* = 6). Homogenisation and further handling of the tissue was performed as previously described in detail^37^. Briefly, the retinae were homogenised in lysis buffer (400 µl T-PER Tissue protein Extraction Reagent/Retina (Thermo Fischer, 78510) using a Precellys 24 homogeniser with 1.4 and 2.8 mm ceramic balls. Afterwards, the retinal homogenate was exchanged into 300 µl phosphate-buffered saline (PBS) using an Amicon 3 kDa centrifugal filter device (Millipore, Billerica, MA, USA). Protein concentration was determined with a BCA protein assay kit (Thermo Fisher Scientific, Rockford, IL, USA) according to manufacturer’s instructions and measured with a Multiscan Ascent photometer (Thermo Fisher Scientific, Rockford, IL, USA) at a wavelength of 570 nm. For the SDS gel protein separation, 2 retinas were pooled (resulting in *n* = 3 for WT and HT) and 50 µg of each pooled sample was loaded per lane and separated with 10-well NuPAGE 12% Bis–Tris minigels (Thermo Fisher Scientific, Rockford, IL, USA) under reducing conditions. In gel tryptic digestion was performed as described in detail previously and SOLAµ^TM^ HRP Spin plates were used for peptide purification prior to LC-MS/MS analysis with a Rheos Allegro Pump downscaling to a capillary HPLC system and coupled to a hybrid linear ion trap-Orbitrap MS system (LTQ Orbitrap XL; Thermo Fischer Scientific, Rockfeld, IL USA). Elution gradient of the HPLC and MS-specific parameters are described elsewhere in detail^38^. Parameters used for elution gradient and MS parameters were applied as described previously^39,40^. Data analysis was performed using Perseus software package version 1.6.5.0^41^. LFQ intensities were log2 transformed and ANOVA was performed. For protein interaction analysis STRING software version 11.0 was used.

### RGC culture

Postnatal day 1 (P1) *Ambra1*^+/gt^ and *Ambra1*^+/+^ littermates were used to isolate RGCs. The eyes were extracted and the retina isolated. The retina was dissociated using the Papain kit from Worthington (PDS LK003150l). Then 25,000 retinal cells were incubated in 96-well plates (Screenstar microplate, Greiner Bio-one, 655866), pre-incubated with poly-l-lysine and laminin, with neurobasal A (ThermoFisher, 21103049) medium containing 2% B27 (ThermoFisher, 17504044), 0.5% gentamicin (Gibco, 15710-049), 0.25% l-glutamine (Gibco, 25030-024). Cells were incubated up to 3 days. Light microscopic pictures were taken after 1 and 3 days in vitro (DIV) and axonal neurites were measured manually in masked pictures. Five images were taken per well (*n* = 15 per time point). DAPI (Sigma, D9542; 1:1000) staining was performed to determine the total number of cells as well as the number of apoptotic nuclei, which were assessed for each condition in a masked manner. Neuronal Class III Beta-Tubulin (TUJ1) (Biolegend, MMS-435P at 1:500) staining was performed to analyse the % of RGCs in the culture.

### Statistics

Prism *Version 8.3.19 was used for statistical analysis. Mann–Whitney tests or Krusak-Wallis ANOVA were performed. *p*-values < 0.05 were estimated to be statistically significant. Graphs were performed with GraphPad Prism (Version 8.3.1) and represent ± SD or ± SEM (indicated in the figure legends).

## Results

### Decreased RGC survival in aged autophagy deficient *Ambra1*^+/gt^ mice

**Fig. 1:**
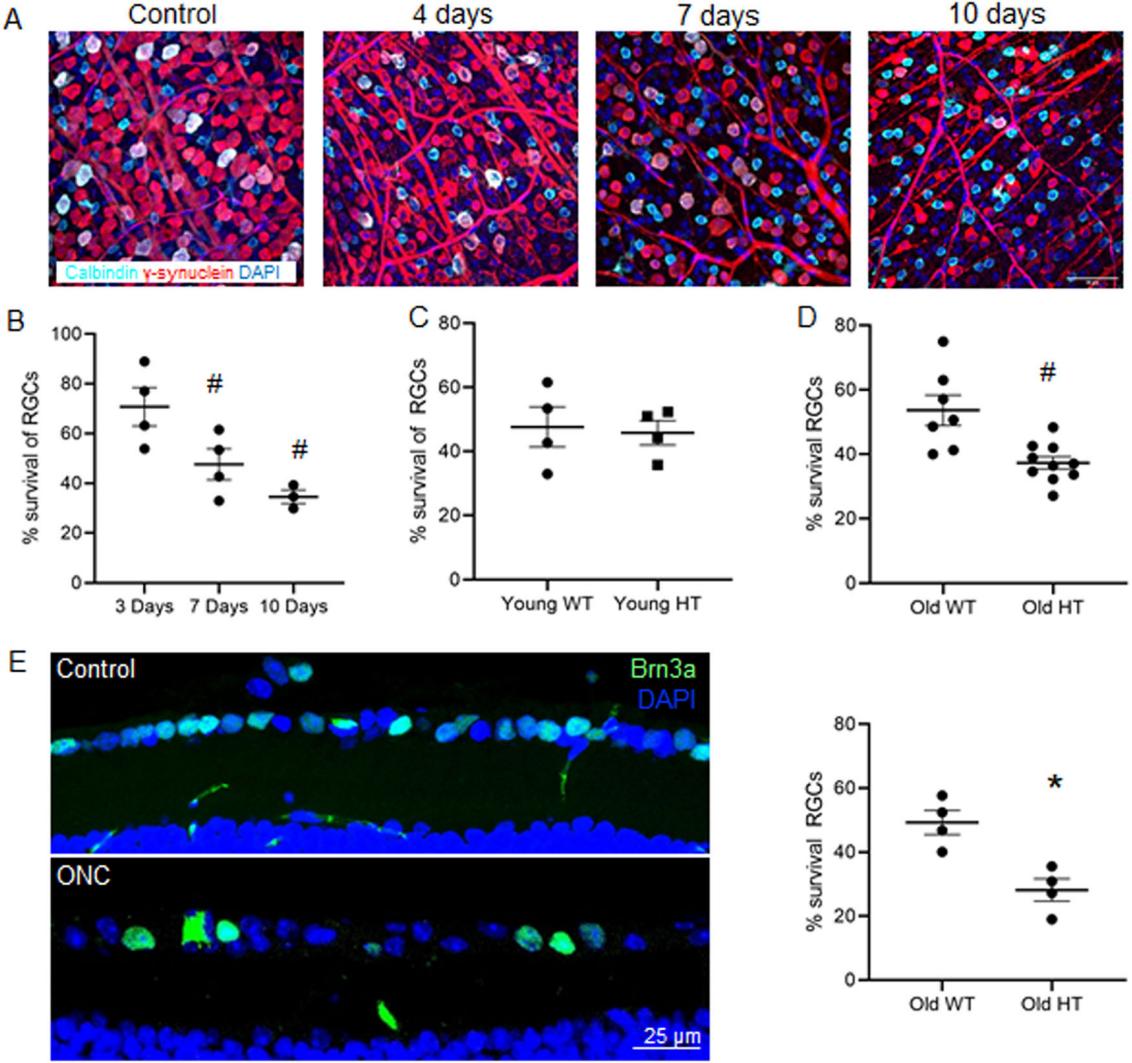
Old *Ambra1*^+/gt^ mice display reduced RGC survival after ONC. **a** Immunostaining of retinal ganglion cells in flat mounts at different time points after ONC showing γ-Synuclein (red) and Calbindin (cyan) staining. **b** % of RGCs throughout a time course up to 10 days after ONC in young Ambra1^+/+^ (WT) animals. The number of RGCs was calculated by subtracting from the amount of γ-Synuclein positive cells minus the displaced amacrine double stained γ-Synuclein and Calbindin cells. The control eyes are represented as baseline value, *n* = 4 animals per group. ^#^*p* < 0.01 in comparison to the corresponding non-injured eye. **c** % survival of RGCs in 3-months-old (young) *Ambra1*^+/+^ and *Ambra1*^+/gt^ mice 7 days after ONC, *n* = 4 animals per group. **d** Graph showing the survival of RGCs in old (13-months-old) Ambra1^+/+^ and Ambra1^+/gt^ mice 7 days after ONC, *n* = 8 animals per group. ^#^*p* < 0.01 **e** % of RGCs stained with Brn3a in cryosections of old *Ambra1*^+/+^ and *Ambra1*^+/gt^ mice 7 days after ONC, *n* = 5 animals per group. Data show mean +/− SEM, *p < 0.05.

The ONC is a model to mimic RGC death through IOP independent optic nerve damage. It therefore relates more to glaucoma without elevated eye pressure than POAG. As NTG has been linked to genes involved in autophagy and mitophagy, and is an age-related disease, we aimed to detect changes in RGC loss after ONC depending on age and autophagy impairment. For this, *Ambra1*^+/gt^ animals and *Ambra1*^+/+^ littermate controls were subject to ONC, as AMBRA1 is involved in autophagy induction and serves as a mitophagy receptor. After ONC, the left eye was assessed at different times point after injury. To determine the number of RGCs, both the right and the left retina were flatmounted and stained with Gamma-Synuclein and Calbindin. Gamma-Synuclein staining serves as robust RGC marker in control and damage conditions^42^, however also stains displaced amacrine cells^43^. Although Gamma-Synuclein staining appears weaker in displaced amacrine cells, and therefore could be distinguished, Calbindin staining was used to identify these cells. Cells only stained for Gamma-Synuclein were counted as RGC. As expected, we found a steady, significant decrease of RGCs after ONC, Fig. 1a. RGC numbers decline after ONC in comparison to the respective control eye, from day 4 until day 10 after ONC, Fig. 1b. Seven days post-ONC was chosen as endpoint for the further experiments. We then compared RGC counts of young and old *Ambra1*^+/+^ and *Ambra1*^+/gt^ 7 days after ONC. When comparing young *Ambra1*^+/+^ and *Ambra1*^+/gt^, we found a significant decrease in RGCs after ONC in both groups but no difference in amount of RGC loss Fig. 1c. In contrast, RGC counts after ONC in the aged animals showed significantly reduced RGC numbers in the *Ambra1*^+/gt^ animals than in the *Ambra1*^+/+^ mice, Fig. 1d. Retinal cryosections and Brn3a staining also showed significantly decreased RGC numbers in *Ambra1*^+/gt^ animals after ONC in comparison to *Ambra1*^+/+^, Fig. 1e. These data show an age-dependent decline of RGC survival in an autophagy deficient model.

### Altered mitochondrial and neuronal proteins in *Ambra1*^+/gt^ retinae

**Fig. 2:**
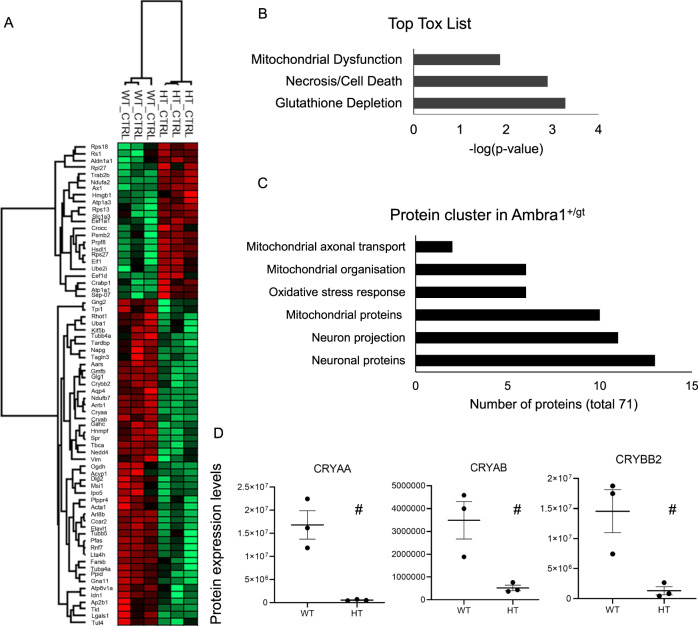
Proteomics of *Ambra1*^+/gt^ vs *Ambra1*^+/+^ retina. **a** Heat map depicting the hierarchical clustering of the differentially expressed proteins in the old *Ambra1*^+/+^ (WT) and *Ambra1*^+/gt^ (HT) animals at baseline. Gene names of the proteins are listed. Green indicated proteins significantly downregulated, red indicated proteins significantly upregulated. **b** Significant toxicological list derived from the IPA analysis of the old *Ambra1*^+/gt^ (HT) vs. *Ambra1*^+/+^ (WT) animals at baseline, after performing Benjamini-Hochberg correction. **c** Relevant protein clusters statistically significantly differently regulated between *Ambra1*^+/+^ (WT) and *Ambra1*^+/gt^ (HT) animals derived from the String analysis (*p* < 0.05). The number of proteins belonging to the clusters is displayed. **d** Alpha-crystallin A-Chain (CRYAA), Alpha-crystallin B-chain (CRYBA) and Beta-crystallin B2 (CRYBB2) protein expressions in *Ambra1*^+/+^ and *Ambra1*^*+/*gt^, ^#^*p* < 0.01.

As we found differences between RGC survival in old *Ambra1*^+/gt^ and *Ambra1*^+/+^ animals, we wanted to explore the molecular players governing this interesting differential RGC survival between these two animal groups. Applying a bottom-up proteomic approach using a HPLC- LTQ Orbitrap XL MS setup, we aimed to understand relevant proteomic changes between old *Ambra1*^+/gt^ and *Ambra1*^+/+^. We identified 1272 proteins in the retinae of the 13-month-old animals, using a false discovery rate <1% as filtering criteria. Seventy-one proteins were significantly differently regulated in *Ambra1*^+/gt^ in comparison to *Ambra1*^+/+^ animals, Fig. 2a. Ingenuity Pathway Analysis (IPA, Quiagen) found changes in glutathione depletion, cell death as well as mitochondrial dysfunction to be among the highest scores, Fig. 2b. 10 of the 71 proteins were mitochondrial proteins, and also 6 of them involved in mitochondrial organisation, 6 proteins were associated with the oxidative stress response, 13 were associated with building neuronal projections and neuronal proteins, 7 of the proteins are associated with eye development, and we also found 2 proteins involved in mitochondrial axonal transport, Fig. 2c. String analysis of these proteins showed a complex protein interaction network with a PPI-value of 2.16e−06, indicating that these proteins have significantly more interactions among themselves than a random set of the same amount of proteins and that these proteins are biologically connected as a group, Supp Fig. 1. Additionally, we found three crystallins (CRYBB2, CRYAA, and CRYAB) to be significantly downregulated in the retinae of the old *Ambra1*^+/gt^ animals, Fig. 2d. IPA revealed phagosome maturation to be one of the most significantly altered canonical pathways (*p*-value:10.0E−04), including significantly decreased ATP6V1A expression, Suppl. Fig. 2. These data suggest alterations in the mitochondrial function, oxidative stress response, and downregulation of crystallins, a family of chaperones that has important cytoprotective functions in the eye^44,45^ and has been linked to the regulation of lysosomal acidification via ATP6V1A.

### Impaired oxidative stress response after ONC in *Ambra1*^+/gt^ animals

**Fig. 3:**
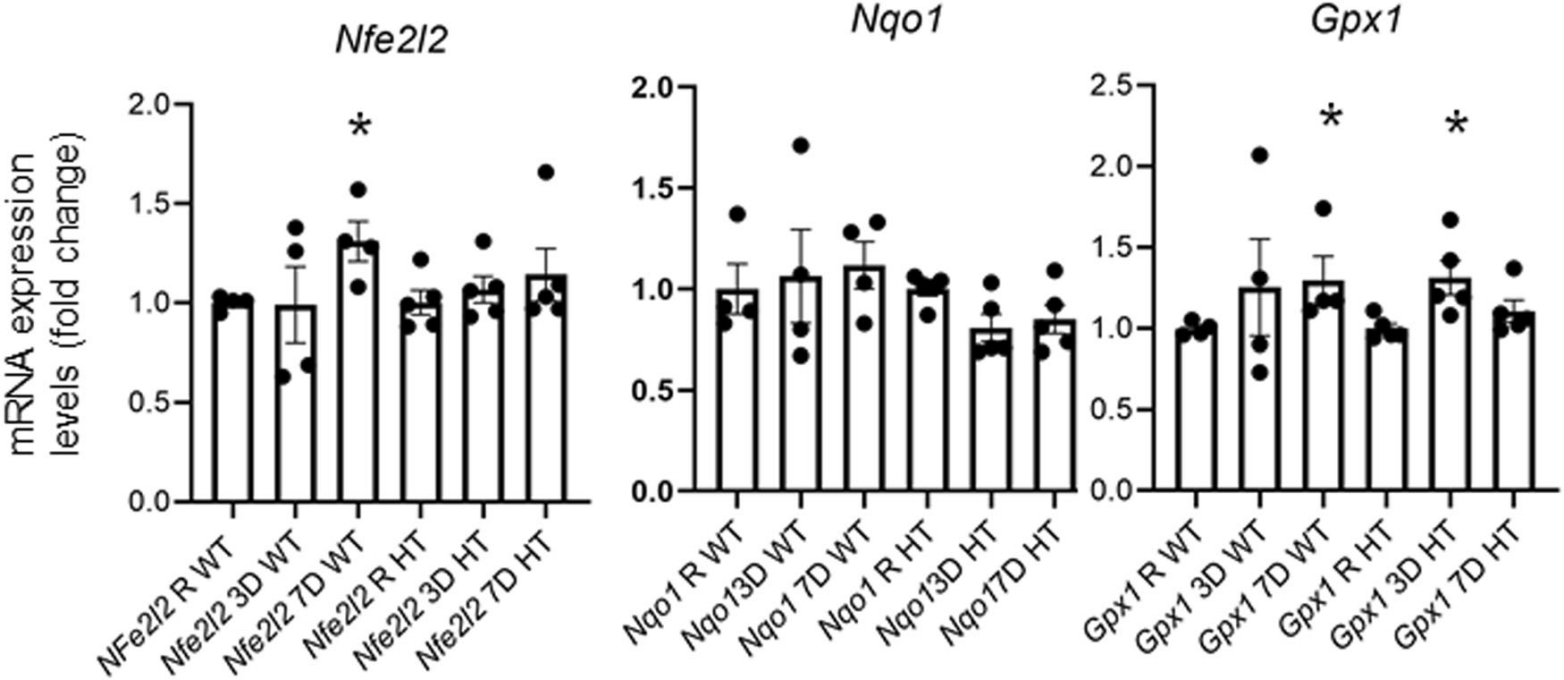
Impaired oxidative stress response in old *Ambra1*^+/gt^ after ONC. mRNA expression levels determined by qPCR of *Nfe2l2*, *Gpx1* and *Nqo1* were measured in old *Ambra1*^+/+^ and *Ambra1*^+/gt^ mice retinas 3 and 7 days after ONC. Data represent mean +/− SEM, *p < 0.05 in comparison to the corresponding control eye, *n* = 4–5 per group.

As oxidative stress and mitochondrial damage were the main pathways altered, we next decided to follow up in this pathway. Given the reported connections between NFE2L2, mitochondria disfunction and oxidative stress response^46,47^,we performed qPCR analysis studying *Nfe2l2* (also known as *Nrf2*), and its downstream factors *Nqo1* (NAD(P)H Quinone Dehydrogenase 1) and *Gpx1* (Glutathione peroxidase 1) in the retinae 3 and 7 days after ONC in *Ambra1*^+/+^ and *Ambra1*^+/gt^ animals compared to the contralateral control eyes. *Nfe2l2* showed a significant increase 7 days after ONC in *Ambra1*^+/+^ animals, whereas the expression levels were not altered in the *Ambra1*^+/gt^ animals, Fig. 3. Last we detected a significant increase of *Gpx1* 3 days after ONC in the *Ambra1*^+/gt^ animals, however lower levels 7 days after ONC, Fig. 3. All these data support our proteomics data indicating that old *Ambra1*^+/gt^ mice have an impaired retinal oxidative stress response after RGC damage.

### Altered mRNA mitophagy regulators levels in *Ambra1*^+/gt^ mice after ONC

**Fig. 4:**
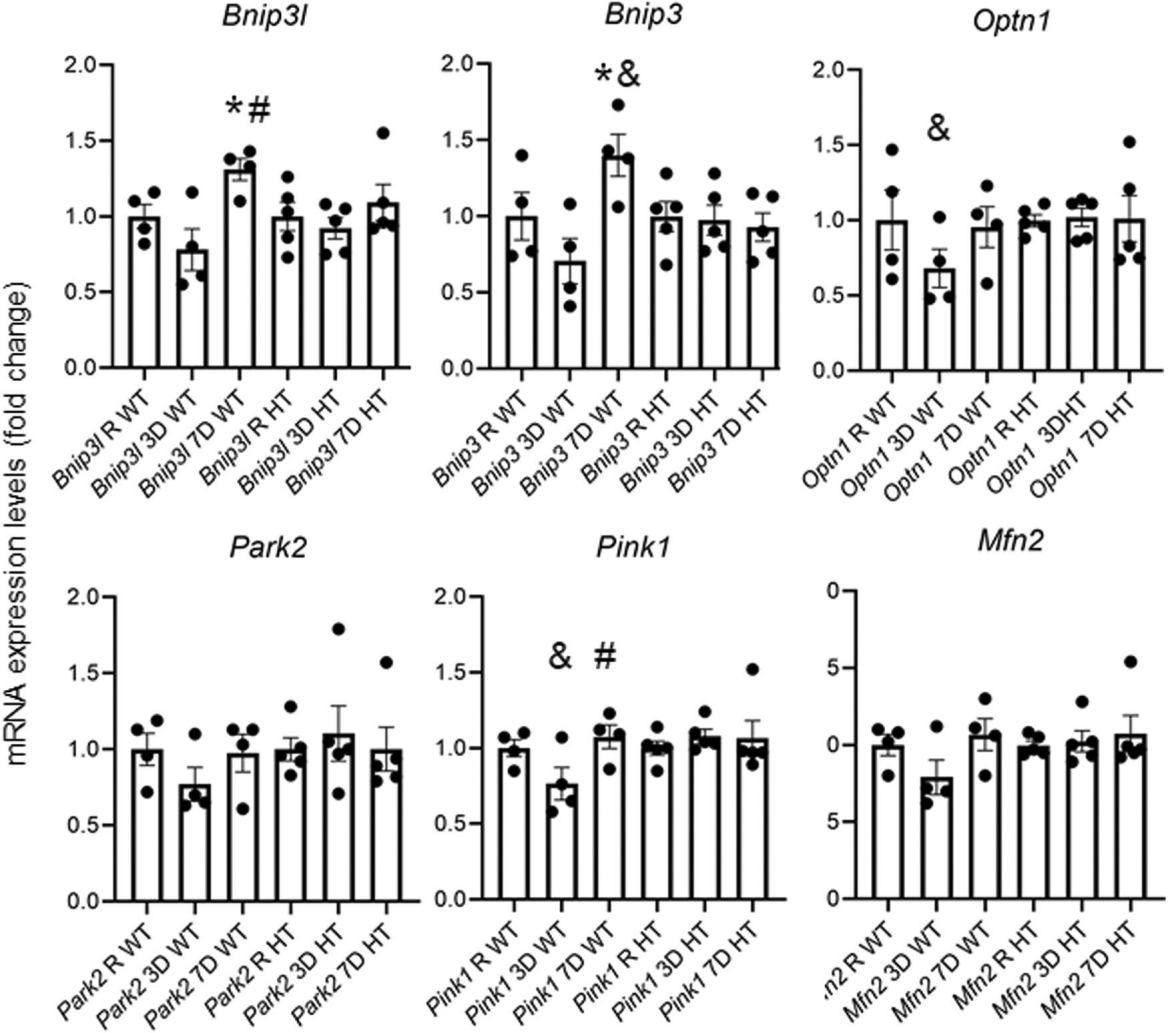
Mitophagy regulator mRNA expression altered after ONC. mRNA expression levels determined by qPCR analysis of mitophagy regulators and receptors *Pink1, Parkin, Bnip3l, Bnip3, Optn*, and *Mfn2* were performed in old *Ambra1*^+/+^ and *Ambra1*^+/gt^ mice of the retinas 3 and 7 days after ONC. Data represent mean +/− SEM, *p < 0.05), *n* = 4–5 animals per group. *p < 0.05 represents the significance against the corresponding control eye, ^#^*p* < 0.05 vs. 3D value of the same genotype, and ^&^*p* < 0.05 vs. same time point in the *Ambra1*^+/gt^ mice.

**Fig. 5:**
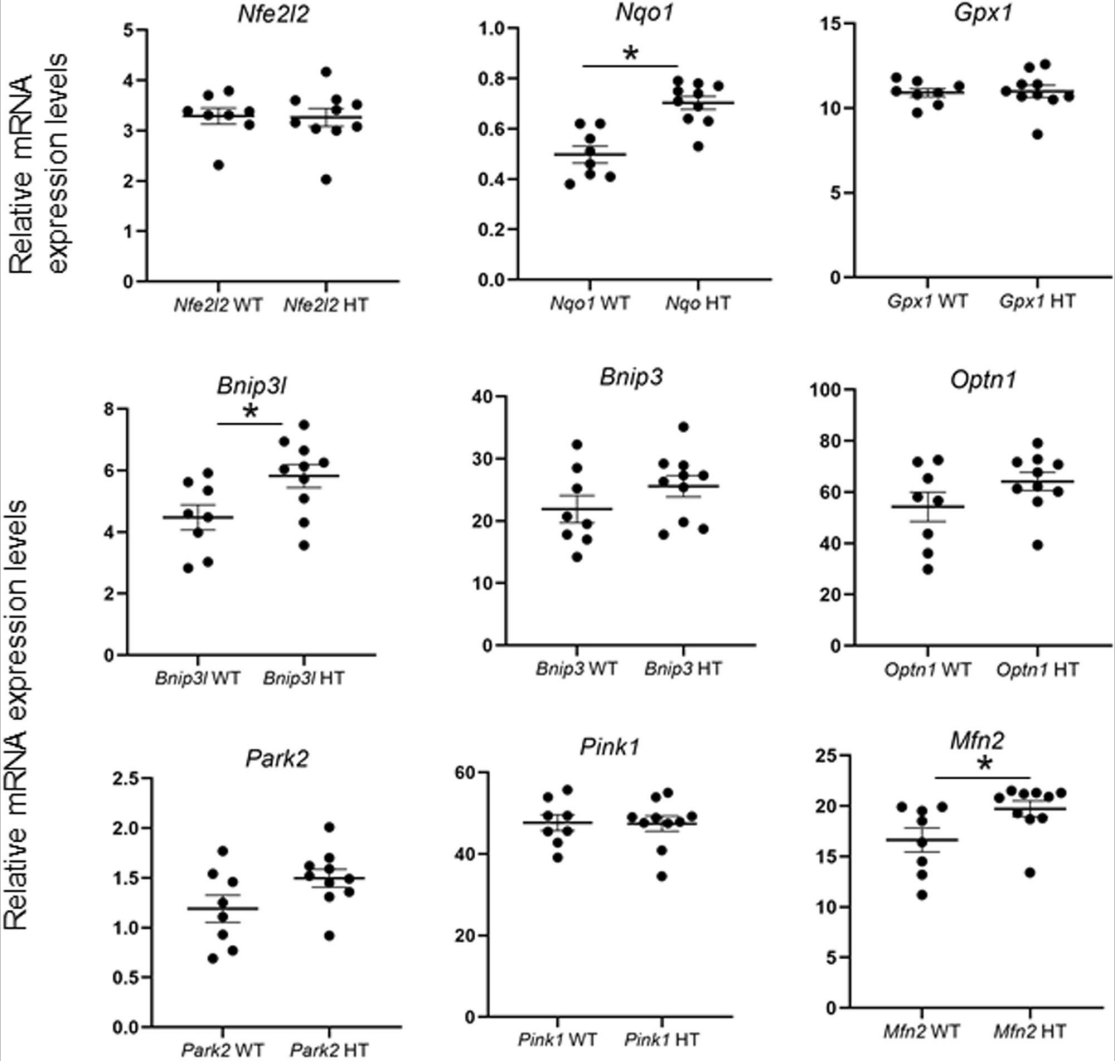
Altered mitochondrial and oxidative stress gene levels in control *Ambra1*^+/gt^ retina. mRNA levels of oxidative stress response genes *Nfe2l2, Gpx1, Nqo1*, and mitophagy receptors and adaptors *Pink1, Parkin, Bnip3l, Bnip3, Optn*, and *Mfn2* were measured in old *Ambra1*^+/+^ and *Ambra1*^+/gt^ mice retinas. The graphs represent the relative mRNA expression levels. Data represent mean +/− SEM, *p < 0.05, *n* = 4–5 animals per group.

Extensive evidences in the literature have found increased oxidative stress markers, such as in the serum of patients, associated to glaucoma^48^ and in animal models of this disease, including ONC^49^. Our data also shows that *Ambra1*^+/+^ animals, but not the *Ambra1*^+/gt^ respond to these levels of oxidative stress by increasing the expression of the master antioxidant regulator *Nrf2*. These observations suggest a loop where increased ROS could exacerbate mitochondrial damage in the absence of an adequate antioxidant response after autophagy deficiency. Another pathway that protects cells against ROS is the elimination of whole damaged mitochondria via autophagy during mitophagy^50,51^. Several lines of evidence suggest that AMBRA1 is a mitophagy receptor that can act in both PINK1/PARKIN dependent and independent pathways^31,32^. While some studies have found mitophagy events in RGCs after optic nerve transection^36^ it is currently unknown how this pathway affects RGCs survival and which mitophagy regulators could be playing a role. Therefore, we decided to assess mRNA expression of several mitophagy regulators after ONC. Seven days after ONC we could detect an increase in *Bnip3l* and *Bnip3* in the *Ambra1*^+/+^ animals, that was not observed in the *Ambra1*^+/gt^, Fig. 4. Conversely, *Ambra1*^+/gt^ did not show any changes of mitophagy regulators 3 or 7 days after ONC, Fig. 4c–f. Other mitophagy regulators such as *Optn1*, *Park2*, *Pink1*, and *Mfn2* showed no changes. Interestingly, when we assessed the mRNA expression in the non-injured eye, we found that *Ambra1*^+/gt^ mice already displayed increased mRNA expression of both *Bnip3l, Nrf2* and *Mfn2* (Fig. 5), suggesting an increased protective response in the absence of axonal damage. This data could then suggest that the *Ambra1*^+/gt^ display an attempt to increase protective pathways which possibly cannot be further upregulated after an additional stress such as axonal damage. We therefore can show, that old *Ambra1*^+/gt^ mice display a misbalance in the oxidative stress regulation as well as mitochondrial proteins in the non-injured retina, which could be leading to a higher vulnerability status of the RGCs after injury.

### Increased apoptosis and decreased neurite growth in *Ambra1*^+/gt^ RGCs

**Fig. 6:**
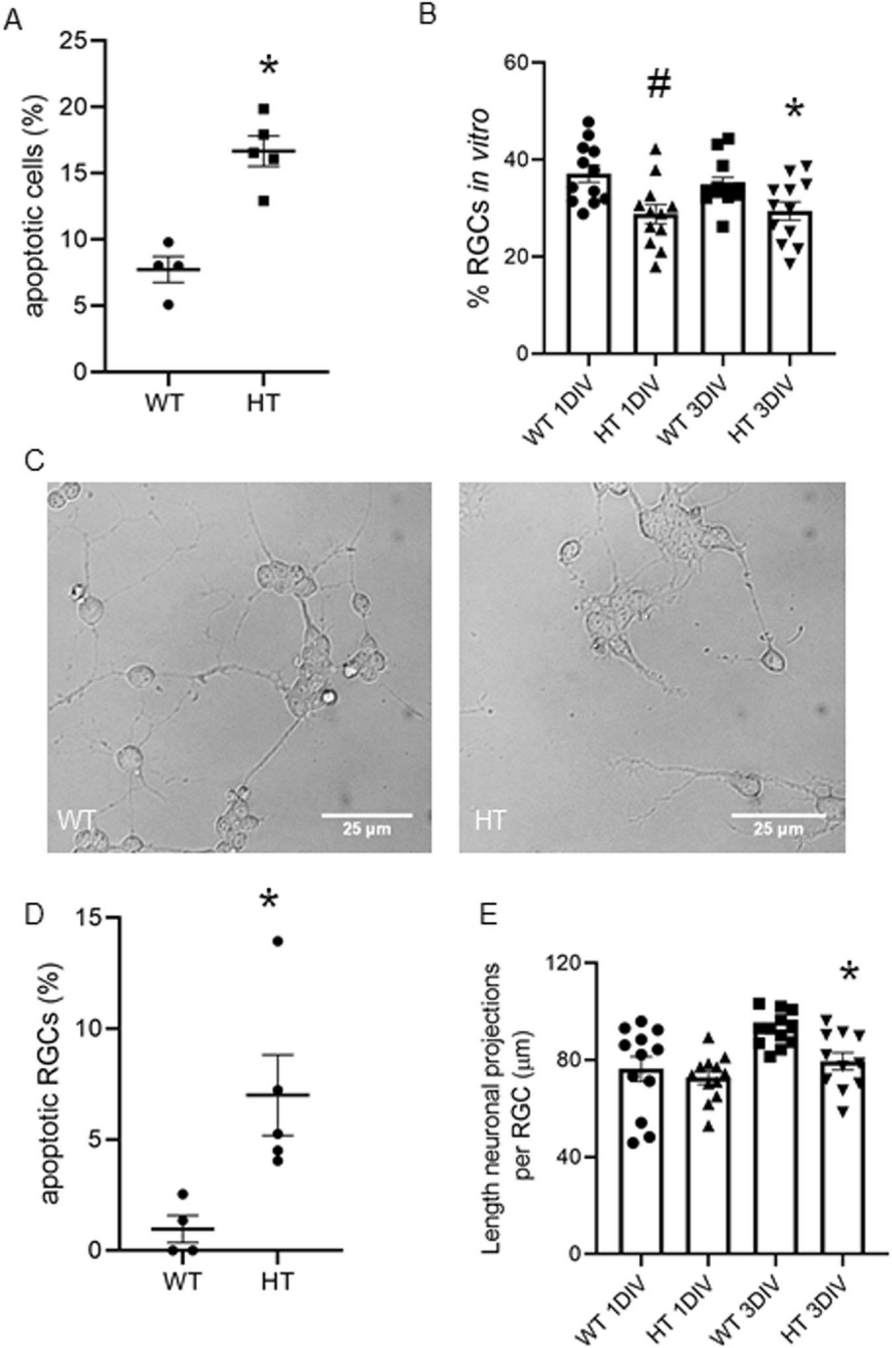
Increased RGC apoptosis and decreased neuronal growth in *Ambra1*^+/gt^ derived RGCs. **a** % of apoptotic cells determined by counting condensed nuclei in *Ambra1*^+/+^ and *Ambra1*^+/gt^ retinal cell cultures (*n* = 4 for WT, *n* = 5 for HT), ^#^*p* < 0.01, referring to comparison with WT, Data represent mean +/− SEM. **b** RGC numbers in culture were determined 1 and 3 DIV using DIC pictures. The graph shows the number of RGCs, *n* = 15. ^#^*p* < 0.01, *p < 0.05. Data represent mean +/− SEM. **c** Representative DIC pictures of the RGCs cultures after 3DIV. The scale bar 25 µm. **d** % of apoptotic RGCs in culture in *Ambra1*^+/+^ and *Ambra1*^+*/*gt^ Apoptotic RGCs were counted using DAPI and TUJ1 staining (*n* = 4 for WT, *n* = 5 for HT), *p < 0.05, in comparison to the corresponding WT value. **e** The length of RGC neurites was measured manually in DIC pictures in *Ambra1*^+/+^ and *Ambra1*^+/gt^. The graph represents the length of neuronal projection that each RGC grows at 1 DIV and 3 DIV, *n* = 15, *p < 0.05, referring to comparison with corresponding WT value, data represent mean +/− SEM.

So far, the field of RGC research is still in need of a robust RGCs in vitro model to study alterations in the context of autophagy or mitophagy deficiency. We used a previously optimised protocol for RGC cultures from neonatal mice to study primary RGCs derived from *Ambra1*^+/gt^ and *Ambra1*^+/+^ mice in vitro. We isolated RGCs from retinas of neonatal *Ambra1*^+/gt^ and *Ambra1*^+/+^ mice and determined their morphology and survival. We observed an increase in the number of apoptotic cells in the *Ambra1*^+/gt^ derived cells in comparison to the *Ambra1*^+/+^ cells, Fig. 6a. Counting the number of RGCs in the culture, we found that *Ambra1*^+/gt^ derived cells had significantly less RGCs than *Ambra1*^+/+^ cultures, Fig. 6b, c. Additionally, the RGCs in the *Ambra1*^+/gt^ cultures displayed significantly more apoptotic nuclei, Fig. 6d. Last, the number of neuronal projections was comparable in both the *Ambra1*^+/gt^ and *Ambra1*^+/+^ RGCs, however the length of outgrowing projections was significantly decreased in the *Ambra1*^+/gt^ derived RGCs, Fig. 6e. Our results demonstrate that primary RGCs derived from *Ambra1*^+/gt^ retinae show a higher vulnerability shown by increased rates of apoptosis. Furthermore, the ability of neurite outgrowth is significantly reduced in *Ambra1*^+/gt^ derived primary RGCs.

## Discussion

In this study we have demonstrated an age dependent decrease in RGC survival after ONC in the *Ambra1*^+/gt^ mice compared to wildtype littermates. This is especially striking as we used middle aged animals (13 months of age), which do not display any apparent phenotype at this age. Mitochondrial alterations with impaired stress related mitophagy induction and altered oxidative stress response could play a role in the increased RGC susceptibility in the old *Ambra1*^+/gt^ animals. This is supported by our proteomic data as well as mRNA transcription for mitochondrial and oxidative stress genes. Our in vitro data additionally suggest a role for AMBRA1 in RGC survival and neurite outgrowth.

We demonstrate increased RGC loss in 13-month-old *Ambra1*^+/gt^ in comparison to *Ambra1*^+/+^ littermates and to the young experimental groups. We can rule out a general age-related decline in RGC survival, as the old *Ambra1*^+/+^ did not show any changes in RGC loss after ONC in comparison to the young *Ambra1*^+/+^. Only few studies have analysed age related RGC susceptibility after ONC however, reduced RGC numbers after ONC has been shown for older animals, such as a study showing increased RGC damage after ONC for 24-month-old mice in comparison to young animals^52^. Regarding *Ambra1*^+/gt^ no studies have been performed analysing the aging-related phenotype, but we did not observe differences in the number of RGCs in non-injured retinas (data not shown). Recent studies, however, have revealed that female *Ambra1*^+/gt^ mice have some autistic features, but these are already apparent in young animals. Some of the analysed changes in the young animals however showed a slightly different characteristic in the middle aged mice, such as the form of induced seizures^53,54^, pointing towards a possible age related change. In our study we used a female to male ratio of 1:1 with an outbred CD1 background, whereas the studies showing autistic features were performed in *Ambra1*^+/gt^ with a C57BL/6N background and observations were only found in the female mice. Another study analysing neurogenesis in adult mice could demonstrate the importance of the *Ambra1/Beclin 1* autophagy pathway in this context^55^. Increased RGC death after a severe ischaemia/reperfusion model, induced by increasing IOP and causing ischaemia of the retina for 60 mins, has been demonstrated previously in the *Ambra1*^+/gt^, the age of the animals was not stated in the paper^56^. None of the studies showed an age-related impact of *Ambra1*^+/gt^. With our results we therefore are the first to demonstrate an age related increase in RGC vulnerability in the context of autophagy deficiency, leading to the conclusion that *Ambra1*^+/gt^ mice could be a perfect model to study age related neurodegenerative diseases in the context of autophagy deficiency.

Our experiments show an altered oxidative stress response in *Ambra1*^+/gt^ animals, which could contribute to increased RGC loss after ONC in the aged *Ambra1*^+/gt^ animals. 8.5% of the proteins significantly differently regulated in the *Ambra1*^+/gt^ animals are involved in the oxidative stress response. Glutathione depletion was the most significantly altered toxicological function pathway found in the IPA analysis in the aged *Ambra1*^+/gt^ retinae, pointing towards dysregulation of this pathway in the retinae of these animals. Glutathione is one of the most prominent intracellular antioxidant systems^57^ that it is known to decline with age and promote stress-induced premature senescence in RPE cells^58^. *Ambra1*^+/gt^ animals show altered oxidative stress response after ONC displaying reduced mRNA *Gpx1* induction in *Ambra1*^+/gt^ animals, and the failure to increase *Nrf2* (*Nfe2l2*) mRNA or to adequately maintain *Nqo1* mRNA levels sufficiently after ONC. GPX1 belongs to the very potent group of hydroperoxide-detoxifying enzymes, protecting the cell against oxidative damage such as offering a first line response for tackling mitochondrial derived ROS^59,60^. Briefly, upon overload of the GPX1 response, ROS accumulate and transcription of antioxidant genes such as the downstream NRF2 genes are activated. Increased *Nrf2* expression promotes RGC survival after ONC and also plays a role in protecting RGCs against IOP related damage^61,62^. Additionally, *Nrf2* knockout mice have also been shown to exacerbate optic neuritis in an experimental autoimmune encephalomyelitis model as well as in an ocular ischaemia and reperfusion model^63,64^. NQO1 is another important antioxidant protein and can transcriptionally be regulated via *Nrf2*, as well as Aryl hydrocarbon receptor (AhR), although this does not necessarily lead to the same expression pattern^65^. Another study analysing *Nqo1* mRNA expression found a slight increase 1 day after ONC, however 4 and 7 days after ONC, the levels were back to baseline^66^. We believe that impaired oxidative stress response plays an important role for higher RGC vulnerability after damage in the aged *Ambra1*^+/gt^ autophagy impaired animals. We furthermore conclude, that non-injured *Ambra1*^+/gt^ animals already have a higher activity of stress response mechanisms, due to autophagy deficiency. These chronically induced pathways could lead to an exhaustion of the system. If additional factors such as age in combination with more severe damage, as performed with the ONC, are present, these pathways fail to protect the RGC.

Mitochondria are the main source of ROS within the cell and mitophagy is not only induced by oxidative stress but also plays a major role in modulating ROS in the cell^67,68^. More specifically, AMBRA1 mediated mitophagy plays an important role in counteracting oxidative stress in neuronal cells^69^. Our results suggest increased mitophagy induction (*Bnip3* and *Bnip3l*) in *Ambra1*^+/+^ animals) 7 days after ONC, whereas those levels are not increased in the *Ambra1*^+/gt^ animals. Increased *Bnip3* levels 7 days have also been demonstrated in other CNS damage models such as in acute CNS ischaemia^70^ and induction of mitophagy via *Bnip3l* showed neuroprotective effects in acute cerebral damage independently of PRKN^71,72^. More generally, rapamycin has demonstrated beneficial effects in the context of acute CNS damage showing increased neuronal survival after spinal cord or brain injury as well as RGC rescue after optic nerve transection and ischaemia/reperfusion^36,56^. Rapamycin is well known to increase autophagy, and also upregulates mitophagy and mitochondrial fission in in vivo CNS damage models^73–76^. Supporting possible increase of mitophagy after ONC, EM pictures of RGCs after optic nerve transection show increased numbers of mitochondria in autolysosomes 6 days after the insult^36^. It is tempting to speculate that increasing mitophagy therefore could possibly have a beneficial role for optic nerve damage and glaucoma.

We can show that primary RGCs derived from postnatal *Ambra1*^+/gt^ animals show less survival and reduced neurite outgrowth in vitro in comparison to *Ambra1*^+/+^ derived RGCs. Eleven of the proteins found to be differently regulated in *Ambra1*^+/gt^ animals are involved in neuron projection and 7 proteins are involved in eye development. AMBRA1 plays an important role in embryonic neuronal development, leading to early embryonic death when totally knocked out^30^. However, *Ambra1*^+/gt^ animals show normal development and no apparent phenotype. Studies have demonstrated the role of AMBRA1 induced autophagy in the development of embryonic olfactory bulb neurons. Using eOBSCs (Stem/progenitor cells from the mouse embryo olfactory bulb) it was demonstrated that *Ambra1*^+/gt^ stem cells grow less and generate smaller neurospheres and less neurons, underlining the importance for AMBRA1 in neurons in a developmental stage^34^.

Our proteomics data showed decreased expression of several crystallin proteins (CRYAA, CRYAB, and CRYBB2) in *Ambra1*^+/gt^ retinae. Alpha-crystallins display chaperone like features, belong to the small heat shock family^77^ and can protect cells from stress induced apoptosis^78^. Alpha-crystallin expression in the retina is increased in various stress conditions such as diabetic retinopathy or glaucoma, aiming to protect the retinal cells from cell death^4,79^. All of the crystallins found in this study are predominantly expressed in RGCs^44^. More importantly, alpha-crystallins show RGC protective effects after ONC^80^ and neuroprotective effects of beta-crystallin B2 have been extensively studied in the context of RGC damage and survival models, as well as RGC axonal outgrowth^45,81^. Additionally, crystallins can also be linked to age related macular degeneration, and altered crystallins lead to impaired lysosomal clearance in the retinal pigment epithelium^82,83^, however they have not been linked to AMBRA1 so far. Studies have been able to link crystallins (CRYBA1 and CRAYAB) to ATP6V1A expression. ATP6Va1 is a subunit of the vacuolar H+ATPase (V-ATPase) necessary for lysosomal acidification and also for autophagy^84,85^. Our proteomics analysis shows decreased ATP6Va1 in *Ambra1*^+/gt^ retinae, as well as other proteins involved in phagosome maturation. Thus, decreased crystallins in *Ambra1*^+/gt^ mice could not only lead to a loss of neuroprotective functions but also to defective lyosomal function and autophagy arrest. In addition, crystallins increase axonal growth in embryonic as well as young RGCs and neurons in vitro promoting neurite and axonal growth^86,87^ and reduced expression of these proteins could be playing a role in decreased RGC neurite outgrowth of *Ambra1*^+/gt^ derived RGCs.

In conclusion, we have shown an age-related increase in RGC susceptibility in the context of autophagy deficiency. Altered mitochondrial and oxidative stress response proteins in control but also after ONC conditions play a role in increased RGC vulnerability. It can be speculated that in control conditions, old *Ambra1*^+/gt^ mice have increased levels of defence pathways such as the oxidative stress response, resulting in an exhaustion of the system that leads to a reduced response after additional stress conditions. Future research should analyse in more detail the relevance of mitophagy and oxidative stress after neuronal damage to evaluate possible therapeutic targets for glaucoma and other related diseases.

## Supplementary information

Figure legends for Supplementary Figures 1 and 2

Supplement Figure 1

Supplement Figure 2
