# Supplementary material for: Age related retinal Ganglion cell susceptibility in context of autophagy deficiency
Source: Cell Death Discov. 2020 Apr 17;6:21. doi: 10.1038/s41420-020-0257-4 (PMC7165178; doi:10.1038/s41420-020-0257-4)
Supplement: Supplementary file 1 — Figure legends for Supplementary Figures 1 and 2 [file 41420_2020_257_MOESM1_ESM.docx]

Supplementary Figure 1:

Protein Interaction network of the proteins significantly differently expressed in Ambra1^+/gt^ (HT) retinae in comparison to Ambra1^+/+^ (WT) was created using the String analysis software. The PPI- Value was 2.16e-06. 10 proteins were mitochondrial proteins (highlighted in red), 6 proteins are involved in mitochondrial organisation (highlighted in lilac), 6 proteins are oxidative stress response proteins (highlighted in yellow) 13 are associated with building neuronal projections and neuronal proteins (highlighted in blue and light green), 7 of the proteins are associated with eye development (highlighted in turquoise), 2 proteins are involved in mitochondrial axonal transport (highlighted in green).

Supplementary Figure 2:

Shows the protein expression changes measured in the Ambra1^+/gt^ (HT) retinae in comparison to Ambra1^+/+^ (WT) involved in the phaogosome maturation pathway. Proteins downregulated are highlighted in green, proteins found in our proteomics analysis but were not significantly regulated in the Ambra1^+/gt^ retinas. The pathway analysis was performed with Ingenuity pathway analysis (IPA) software, the graph was also designed with IPA.
